# Supplementary material for: A high-resolution mRNA expression time course of embryonic development in zebrafish
Source: eLife. 2017 Nov 16;6:e30860. doi: 10.7554/eLife.30860 (PMC5690287; doi:10.7554/eLife.30860)
Supplement: Supplementary file 6. [file elife-30860-supp6.zip › biolayout-clusters-files/Cluster081-genes.html]

Cluster081


# Cluster081: Genes

| Ensembl ID | Gene Name | Chr | Start | End | Biotype |
| --- | --- | --- | --- | --- | --- |
| ENSDARG00000069335 | BCL6B | 7 | 19765239 | 19776199 | protein\_coding |
| ENSDARG00000056090 | capza1b | 8 | 27788806 | 27808665 | protein\_coding |
| ENSDARG00000035904 | crabp1b | 7 | 48047161 | 48069974 | protein\_coding |
| ENSDARG00000011956 | dlx4a | 3 | 34540568 | 34551913 | protein\_coding |
| ENSDARG00000024537 | emilin1a | 20 | 34942793 | 34962152 | protein\_coding |
| ENSDARG00000039949 | fibina | 25 | 34997620 | 34998470 | protein\_coding |
| ENSDARG00000034572 | gpr143 | 9 | 55466707 | 55501226 | protein\_coding |
| ENSDARG00000071465 | lrrc39 | 22 | 16282180 | 16285932 | protein\_coding |
| ENSDARG00000042690 | s1pr1 | 22 | 16012225 | 16015973 | protein\_coding |
| ENSDARG00000077852 | samd11 | 23 | 23094158 | 23197350 | protein\_coding |
| ENSDARG00000071378 | si:dkey-78l4.14 | 22 | 19522498 | 19527825 | protein\_coding |
| ENSDARG00000071586 | tgfbi | 14 | 26212936 | 26238399 | protein\_coding |
| ENSDARG00000045367 | tuba1b | 23 | 35614754 | 35617716 | protein\_coding |
| ENSDARG00000076010 | twist1b | 16 | 19926724 | 19928330 | protein\_coding |
| ENSDARG00000042961 | zgc:100920 | 8 | 23071916 | 23082308 | protein\_coding |
